# Effect of Oral Prophylactic Measures on the Occurrence of Pre-Eclampsia (OP-PE) in High-Risk Pregnant Women: A Cluster Randomized Controlled Trial

**DOI:** 10.3390/mps4030061

**Published:** 2021-09-05

**Authors:** Aida Kanoute, Jocelyne Gare, Nicolas Meda, Stephane Viennot, Paul Tramini, Laurie Fraticelli, Florence Carrouel, Denis Bourgeois

**Affiliations:** 1 Public Health Service, Department of Dentistry, Cheikh Anta Diop University, Dakar 10700, Senegal; aida.kanoute@gmail.com; 2 Health, Systemic, Process, UR 4129 Research Unit, University Claude Bernard Lyon 1, University of Lyon, 69008 Lyon, France; jvgare@yahoo.fr (J.G.); stephane.viennot@univ-lyon1.fr (S.V.); laurie.fraticelli@univ-lyon1.fr (L.F.); florence.carrouel@univ-lyon1.fr (F.C.); 3 Public Health Laboratory (LASAP), ED2S Doctoral School of Sciences and Health, University Joseph Ki Zerbo, Ouagadougou 7021, Burkina Faso; nicolas.meda@gmail.com; 4 Department of Public Health, Faculty of Dental Medicine, University of Montpellier, 34090 Montpellier, France; paul.tramini@orange.fr

**Keywords:** periodontitis, pregnancy outcome, Pre-eclampsia, prophylaxis, oral hygiene, risk factors, microbiota, placenta, dysbiosis, *Porphyromonas gingivalis*, inflammation

## Abstract

Pre-eclampsia (PE), a pregnancy-specific hypertensive disorder, characterized by the development of placental endothelial dysfunction, remains a major source of maternal and perinatal morbidity and mortality, especially in low- and middle-income settings. Periodontal disorders during pregnancy, and particularly periodontal pathogens, may be related to the risk of PE. Standard oral hygiene methods, based mainly on the joint use of toothbrushes and interdental brushes, reduce periodontal inflammatory risk and modulate the dysbiosis of the oral microbiome. The aim of this trial is to compare the PE outcomes in high-risk pregnant women receiving oral prophylactic measures to a control group. This trial is designed as a two-arm, parallel, cluster randomized controlled trial with the antenatal obstetric clinic as the unit of randomization and an allocation ratio of 1:1. The pregnant women will be included at 3 months of pregnancy and will be followed throughout the pregnancy. The primary outcome measure will be the incidence of PE from a baseline during the pregnancy. Secondary outcomes measures will include changes from the baseline in quantification of the pathogenic bacterial load of the interdental microbiota, the severity scores of periodontal indicators, and the incidence of adverse perinatal outcomes. This trial should demonstrate that the implementation of daily oral hygiene reduces oral dysbiosis, the incidence of periodontal disease, and the risk of PE.

## 1. Introduction

Pre-eclampsia (PE) remains a major source of maternal and perinatal morbidity and mortality, especially in low- and middle-income settings [1]. PE is a pregnancy-specific hypertensive disorder that is characterized by the development of placental endothelial dysfunction resulting in, among the most common diagnostic features, concomitant hypertension and proteinuria that may evolve into organs with fluctuating clinical specificities [2,3]. Obesity, chronic hypertension, metabolic or cardiovascular risks, and severe anemia, predisposing to endothelial dysfunction as part of an excessive systemic inflammatory response, have been found to be the highest risk factors of PE [4,5,6]. However, the prediction and prevention of PE is not possible, as the multifactorial pathogenesis of the different types of PE is not clearly identified [7]. The analysis of the alteration of placental microbiome is a possible therapeutic approach in the prevention and management of PE [8]. As an early signal of a future increase in cardiovascular and metabolic diseases, PE may warrant both educational and lifestyle interventions to improve perinatal outcomes [9,10].

In recent decades, numerous studies have shown that periodontal disease (PD) has contributed to prematurity and low birth weight [11,12,13]. PD is a chronic, multifactorial, non-communicable disease and inflammatory disease of polymicrobial origin resulting from an increase in pathobionts in the microbiota [14]. Maternal periodontopathogens may be linked to placental damage and premature pathogenesis [15], as well as the association between the presence of pathogenic oral bacteria in the placenta and adverse pregnancy outcomes [16]. PD may lead to systemic complications including pregnancy complications [17]. Current studies indicate that periodontal disorders during pregnancy, and particularly periodontal pathogens, may be related to the risk of PE [18]. Conversely, there is limited emerging evidence that pregnancy infers dysbiosis in the maternal oral microbiome [19].

Standard oral hygiene methods, based mainly on the joint use of toothbrushes and interdental brushes, reduce periodontal inflammatory risk and modulate the dysbiosis of the oral microbiome [20,21,22]. Individual oral prophylaxis could impact bloodstream entry of periodontal pathogenic bacteria such as *Porphyromonas gingivalis*, whose action on the placental endothelium is scientifically validated [23]. This periodontal pathogen is the most common microorganism present during bacteremia in patients with periodontitis [24,25], with the capacity to cause inflammation even if present in insignificant quantities [26]. It is also the most common microorganism in the amniotic fluid and placental tissue associated with adverse pregnancy outcome [27,28].

The purpose of this trial is to compare the PE outcomes in high-risk pregnant women receiving oral prophylactic measures to a control group. One specific question is addressed as follows according to the PICO principles: In high-risk pregnant women (Population), do oral prophylactic measures (Intervention) have a beneficial effect on PE parameters (Outcome) compared to pregnant women without any specific oral prophylactic measures (Control)?

## 2. Material and Methods

### 2.1. Trial Design

**Figure 1:**
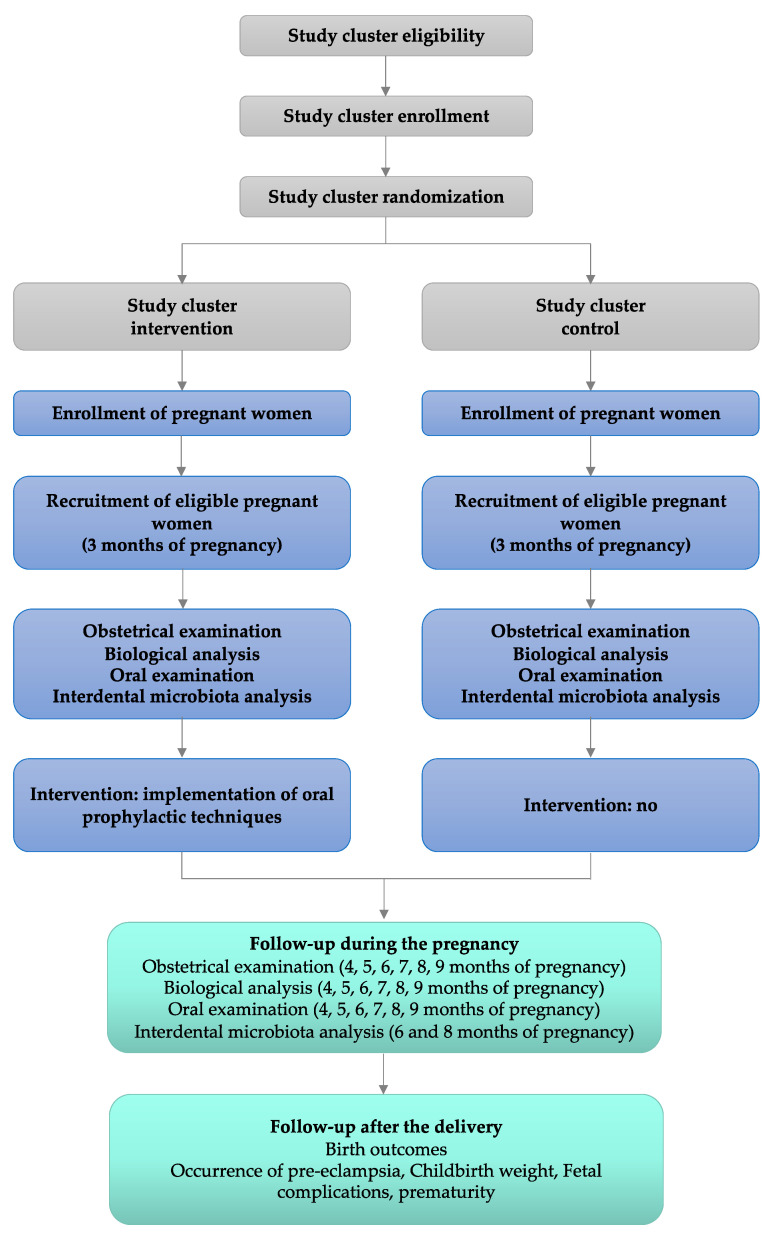
Flow chart diagram.

This study protocol is reported according to the Standard Protocol Items: Recommendations for Interventional Trials (SPIRIT) guidelines [29]. This trial is designed as a two-arm, parallel, cluster randomized controlled trial with antenatal obstetric clinic as the unit of randomization and an allocation ratio of 1:1 (Figure 1). 

### 2.2. Study Setting

This study will be conducted within the establishments of the National Hospital Center of Dakar, Senegal, and of Ouagadougou, Burkina Faso.

### 2.3. Study Population

#### 2.3.1. Eligibility Criteria

The obstetrics clinics of the institutions (clusters) will be selected under the general principle that the attending population in each country should be similar with regard to socio-demographic factors of participants planning to deliver in hospitals. Criteria for clinic selection will be: (i) clinics in the public system, (ii) Each clinic’s ability to include at least 150 new pregnant women over a period of no more than 6 months, (iii) access to clinic is the reference site for all high-risk women for PE, and (iv) intervention and control clinics will be in the same geographical area but serving distinct neighborhoods.

The individual eligibility criteria will be: (i) pregnant woman, (ii) women from sub-Saharan Africa, (iii) aged from 18 to 35 years old, (iv) nulliparous at the time of the obstetrical consultation, (v) up to 12 weeks pregnant, (vi) acceptance of the terms and conditions of the study, and (viii) signature of the informed consent form.

#### 2.3.2. Exclusion Criteria

The exclusion criteria will be pregnant women with: (i) fetal distress, (ii) congenital uterine and vaginal abnormalities, (iii) infectious or systemic diseases such as HIV, tuberculosis, candidiasis, cancers, hemopathies, (iii) premature termination of pregnancy for medical reasons, (iv) periodontal lesions of stage II, III, IV (i.e., PD ≥ 4 mm, and/or CAL ≥ 4 mm), generalized (>30% of sites), (v) history or treatment of PD, (vi) a course of dental or orthodontic treatment, (vii) absence of the 4 premolar–molar pairs, (viii) less than 20 natural teeth, excluding third molars, (ix) medication affecting the gum and/or oral mucosa, (x) regularly using interdental brushes and/or dental floss and/or mouthwash, and (xi) unable to answer questions or non-cooperative.

### 2.4. Interventions

The intervention will be the implementation, or not, of oral prophylactic techniques. The control group will continue its usual oral hygiene practice. For the study group, the oral prophylactic intervention will consist of provision of specific package including soft-bristled manual toothbrush, toothpaste, and a kit of calibrated interdental brushes (IDBs) (Curaprox CPS; Curaden) of sizes corresponding to the diameter of their interdental spaces. The participants will be instructed to brush their teeth twice-daily and to realize daily interdental brushing until delivery. The instructions for the use of the toothbrush and IDBs comprised verbal instructions supported by practical demonstration. The first use of the material will be conducted under the supervision of a qualified public health professor.

Our research is a pragmatic study that does not want to interfere with the organization of care provided during obstetrical visits. In the public health systems, except for very punctual dental emergencies, no periodontal care is suggested or provided. 

### 2.5. Outcomes

#### 2.5.1. Primary Outcome Measures

Primary outcome measure will be the incidence of PE from baseline during the pregnancy. PE is defined as diastolic pressure >90 mm Hg after two 4 h intervals or >110 mm Hg once; systolic pressure >140 mm Hg after two 4 h intervals or >160 mm Hg once, after 20 weeks of gestation in combination with proteinuria 2+ or more; >300 mg/24 h, >500 mg/L, or urinary protein/creatinine ratio >0.034 g/mmol.

#### 2.5.2. Secondary Outcome Measures

Secondary outcomes measures will include changes from baseline in:

- Quantification of the pathogenic bacterial load of the interdental microbiota.
- Severity scores of periodontal indicators: pocket probing depth (PPD), clinical attachment level (CAL), gingival index (GI), plaque index (PI), bleeding on interdental brushing index (BOIB).
- Incidence of adverse perinatal outcomes (low birth weight and preterm delivery).

### 2.6. Participant Timeline

The schedule of visits will overlap with the recommended follow-up schedule for pregnant women. The participation timeline of the trial is presented in the Table 1.

#### 2.6.1. Pre-Screening and Assessment for Eligibility

Participants will be screened at their first prenatal visit. If they meet the eligibility criteria, the study will be presented to them. If they agree to participate and consent to be screened, the inclusion visit will be scheduled at 3 months of pregnancy.

#### 2.6.2. Inclusion, Prenatal Examination, Oral Examination, and Baseline Interdental Microbiota Sampling (T1)

Participants will sign the informed consent form. A clinical obstetric and oral examination, followed by interdental microbiota sampling, will be performed for all participants.

- Prenatal clinical examination

The obstetric clinical examination will include the measurement of weight, height, blood pressure, and the uterine height of the participants.

- Biological analysis

The biological analysis will determine the level of red blood cells, white blood cells, neutrophils, C-reactive protein, creatinine, blood sugar, uremia, and proteinuria.

- Oral clinical examination and sampling of the interdental microbiota

Two calibrated periodontists per site, properly trained before the beginning of the study, and who will be blinded regarding the group category at the time of the evaluation, will perform the periodontal assessment of all patients. Calibration of examiners will include a group of 32 experienced dentists. Intraclass correlation coefficients for PD and CAL will be calculated at the site level. The intra- and inter-examiner coefficients for CAL will range between 0.80 and 0.85, and between 0.75 and 0.85 for PD, respectively.

For all participants, the same four interdental sites (15–16, 25–26, 35–36, and 45–46) will be assessed; the interdental spaces between molars and premolars are the most susceptible to gingival inflammation [30,31]. The interdental diameter will be determined using a graduated conical probe—the CURAPROX IAP calibration probe (Curaden, Kriens, Switzerland) [32]. The working portion includes colored bands from the tip to the base corresponding to IDBs by increasing diameter. The largest section of each colored band corresponds to the cleaning efficiency diameter of the respective brush. This will make it possible to select the calibrated IDB (Curaden) appropriate to the diameter of the interdental space. Each previously selected tooth will be isolated using sterile cotton rolls, and the interdental biofilm will be removed using this sterile IDB. For each sample, the IDBs will be placed in 1.5 mL sterile microcentrifuge tubes and stored at 4 °C until further analysis.

The BOIB [33] will be evaluated for the four interdental sites (15–16, 25–26, 35–36, and 45–46). This corresponds to the bleeding response to the horizontal pressure applied in the interdental space by a calibrated IDB. After 30 s, bleeding at each gingival unit will be recorded (0 = absence of bleeding after 30 s, and 1 = bleeding after 30 s). Then, interdental diameters and the BOIB will be evaluated for all other interdental sites.

Periodontal assessment, including first the GI and, second, the PI, will be performed. The dental plaque will be measured with the Löe and Silness plaque index, for which scores are as follows: 0 = no plaque, 1 = invisible plaque deposit but can be removed with a curette, 2 = plaque deposit covering the cervical 1/3, 3 = abundant plaque deposit (more than the cervical 1/3). Gingival inflammation will be evaluated with the Löe and Silness gingival index, the scores of which are as follows: 0 = healthy gingiva, 1 = erythematous gingiva not bleeding on probing, 2 = erythematous gingiva bleeding on probing, 3 = erythematous gingiva bleeding spontaneously.

PPD and CAL will be measured according to the criteria established by the consensus report of the 2017 World Workshop on the Classification of Periodontal and Peri-Implant Diseases and Conditions. These were based on stages defined by severity according to the level of interdental clinical attachment loss, and tooth loss, as well as the complexity, extent, and distribution, and incorporating a full mouth examination at six sites on each permanent tooth [34]. The initial stage should be determined using CAL.

- Analysis of the interdental microbiota

Total DNA will be isolated from the interdental brushes using the QIAcube HT Plasticware and Cador Pathogen 96 QIAcube HT Kit (Qiagen, Hilden, Germany), according to the manufacturer’s guidelines. Quantitative real-time PCR will be carried out for total bacterial count and for 6 pathogens: Aggregatibacter actinomycetemcomitans, *Porphyromonas gingivalis*, Tannerella forsythia, Treponema denticola, Prevotella intermedia, and Fusobacterium nucleatum. 

#### 2.6.3. Follow-Up Visits

Visits will be made according to the pregnant woman’s monthly follow-up schedule (Table 1). Obstetric clinical examination, biological analysis, and oral clinical examination will performed at each visit. Interdental sampling will be realized for all participants at 6 and 8 months of gestation. 

Participants in the intervention group will be asked about compliance with the study treatment (control of hygiene methods and techniques, and acceptance).

#### 2.6.4. Delivery Data Collection

Following delivery, clinical data will be collected from medical records: length of gestation, occurrence of PE, birth weight of the child, and fetal complications.

### 2.7. Sample Size

Reduction in PE will be considered the primary outcome variable, and an estimate of the mean difference in PE reduction will be used to calculate the sample size. The primary acceptance criterion will be the PE effect of the evaluated medical devices showing at least a moderate effect of 20%.

To show a reduction in PE from 20% to 12% (with alpha = 5%, beta = 80%), coefficient of intraclass correlation of 0.03 and in a unilateral proportion test, we initially calculated 440 subjects per group. Assuming a potential dropout rate of 10%, 880 participants were determined as the target for patient inclusion.

### 2.8. Recruitment

OP-PE trial will recruit 55 pregnant woman from each of the 16 clinical sites for a total of 880 subjects.

To identify participants, at the first visit for a pregnancy diagnosis, the physician will suggest that women who are less than 12 weeks pregnant, between the ages of 18 and 35, and who have never been pregnant before, participate in the study. If she responds positively, she will receive a trial information sheet and a consent form, and an inclusion visit will be scheduled by a qualified dental personnel. The total number of patients screened and recruited will be tracked. Patient volunteers will receive financial compensation.

### 2.9. Assignment of Interventions

#### 2.9.1. Allocation

In this conventional (parallel) cluster randomized trial, antenatal care clinics will be randomized to either the intervention or control arm at the start of the trial and remain in that arm for the duration of the study [35]. The allocation schedule for randomly assigning clinics will be computer generated, and stratified by study site and clinic characteristics at a central location (UR4129 Research Unit, University Lyon, France) by the study officer of the research using e-CRF Voozalyon 1.3 (Voozanoo, Caluire, France). Once the local investigators are operational for the implementation of the training seminars for the clinic staff, the treatment allocation will be sent by email by the study officer to the principal investigator of the selected site. Eligible adults per site will be included in the study in the order of inclusion until the number of subjects per site specified in the protocol is reached.

#### 2.9.2. Blinding

There will be a separation between the assignment generator and those carrying out the assignment. Implementation of the intervention will be performed by personnel not involved in the data collection. Clinicians, clinical research associates, and statisticians will be blinded regarding the group category at the time of the evaluation and analysis. Identification codes will be retained by the study monitor and will not be opened until the end of the study.

### 2.10. Data Collection, Management, Analysis

#### 2.10.1. Data Collection Methods

Using e-CRF in each site, a unique identification code will be assigned to each subject. All documents, forms, and data files will be labeled with this identification code. Each subject will be counted in the screening and enrollment record. Data from study participants will be collected in the form of anonymized case report forms. The investigator will complete the e-CRF. The investigator can enter and save the collected data in the study computer. The monitor will be informed that the CRF has been fully completed and that no changes have been made. The data can be verified by the monitor and used for internal evaluations. The central study officer will have permanent access via the e-CRF, throughout the study, to the study computers of the 16 different study sites.

The data collected and the questionnaires will be archived for at least 10 years, personal data such as names, addresses, and birthdays will not be kept.

#### 2.10.2. Data Management

Personal and experimental data will be analyzed without any personal characteristics using a unique identification code. Subjects can be traced through the subject re-identification record, which will be kept only at the clinical investigation site. Signed informed consent forms will be retained only at the clinical investigation site.

#### 2.10.3. Statistical Methods

The final analysis of the data will be based on intention-to-treat and will be performed with the STATA MP 16.0 software (College Station, TX 77845, USA). All principal statistical analyses will be based on the clusters as allocated in the randomization [36]. Intention-to-treat analyses will be performed on the imputed data from all patients using multiple imputation by chained equations, based on a Monte Carlo–Markov chain algorithm under missing at random data hypothesis. Unilateral analysis to study the superiority of intervention over control group will be programmed. Data entry and preliminary checks for error and internal validity will be carried out at each study site, with corresponding statistical analyses conducted at a central level. A series of explanatory analyses will also be conducted in which clinics and/or individuals deviating from the protocol will be excluded. Additionally, per-protocol analysis using the same methodology as the intention-to-treat analyses will be performed based on participants with a complete set of outcome data at endpoint.

The main analytical technique used to assess the effect of intervention on the primary outcome will be an application of the Mantel–Haenszel test adapted to the cluster randomization design. Baseline tables will be created comparing the intervention groups with respect to both cluster-level and individual-level risk factors. 

Groups will be compared as at randomization. The primary endpoint will be estimated by the Kaplan–Meier method (survival analysis) and comparison of the different groups by the Log Rank test (*p* < 0.05). A multivariate analysis by a Cox regression model will be used to explore the relationship between the risk function associated with PE and the intervention after adjustment for site, amenorrhea at inclusion, gestational age, maternal age, and periodontal parameters at inclusion. Continuous outcome variables will be analyzed using mixed model regression, with the clinic status treated as a random effect. Analyses will also be conducted to explore the possibility of an interaction effect between intervention and clinic size. All statistical analyses performed will account for the within-clinic correlation that would invalidate the application of standard statistical methods. The results will be communicated according to CONSORT guidelines.

Secondary analyses will also be performed comparing, as at randomization, the: (i) evolution of the pathogenic bacterial load of the interdental microbiota in the pregnant women intervention group compared to the control group, (ii) evolution of clinical signs of periodontal conditions in the pregnant women intervention group compared to the control group, and (iii) effect of individual oral prophylaxis during pregnancy on the risk of adverse perinatal outcomes. For dental clinical outcomes, mean and median values of clinical parameters for all oral, lingual/palatal, and proximal sites will be calculated per patient. Variations between the first and follow-up visits will be calculated.

Microbiological variables will be presented as total anaerobic counts, frequency of detection of target pathogens, counts of each pathogen studied, and proportions of each pathogen in the total microbiota. Total anaerobic counts will be log-transformed to match a normal distribution. 

The Shapiro–Wilk goodness-of-fit test and box plots will be used to determine the normal distribution of quantitative variables. Differences between groups at baseline and at follow-up visits will be determined by paired t-test or Mann–Whitney U tests for quantitative outcomes. In addition, clinical variables will be compared to repeated measures by ANOVA with Bonferroni post hoc correction controlling for visit for within-group comparisons, group (intervention or control) for between-group comparisons, and interaction between amenorrhea time and group. 

Results will be considered statistically significant at *p* < 0.05.

### 2.11. Ethics Statement

The protocol and design of this study have been endorsed by ethical and regulatory authorities and will be carried out in conformance with the Declaration of Helsinki. The ethical committee of Ougadoudou (Burkina Faso) approved the protocol on 10 March 2021 (2021-03-077), and the ethical committee of Dakar (senegal) approved it on 8 June 2021 (000086/MSAS/CNERS/SP). This study was registered at ClinicalTrials.gov (identification number NCT04989075).

## 3. Expected Results

As the conditions for professional prophylaxis or other types of oral health care are not practically provided in the specific context of the public system, our work will support the need for a community-based approach with a willingness to include individual prophylaxis as part of the overall primary health care. The implementation of evidence-based, individual preventive care linked to behavioral changes in oral hygiene quality will reduce PE. This comprehensive trial is a very important topic to evaluate novel strategies for preventive approaches in PE.

## Figures and Tables

**Table 1:** Participation timeline of the trial.

Date of Pregnancy Procedures/Visits	Timeline							
	T0	T1	T2	T3	T4	T5	T6	T7
		3 Months *	4 Months	5 Months	6 Months	7 Months	8 Months	9 Months
Pre-screening	X							
Eligibility screening	X							
Informed consent	X							
Obstetric examination	X	X	X	X	X	X	X	X
*weight*	X	X	X	X	X	X	X	X
*blood pressure*	X	X	X	X	X	X	X	X
*uterine heigh*	X	X	X	X	X	X	X	X
Biological analysis	X	X	X	X	X	X	X	X
*red blood cells*	X	X	X	X	X	X	X	X
*white blood cells*	X	X	X	X	X	X	X	X
*neutrophils*	X	X	X	X	X	X	X	X
*C-reactive protein*	X	X	X	X	X	X	X	X
*creatinine*	X	X	X	X	X	X	X	X
*blood sugar*	X	X	X	X	X	X	X	X
*uremia*	X	X	X	X	X	X	X	X
*proteinuria*	X	X	X	X	X	X	X	X
Oral examination	X	X	X	X	X	X	X	X
*pocket probing depth*	X	X	X	X	X	X	X	X
*clinical attachment loss*	X	X	X	X	X	X	X	X
*plaque index*	X	X	X	X	X	X	X	X
*gingival index*	X	X	X	X	X	X	X	X
*bleeding on interdental brushing*	X	X	X	X	X	X	X	X
Interdental microbiota analysis		X			X		X	

* Date of pregnancy.

## Data Availability

No new data were created or analyzed in this study. Data sharing is not applicable to this article.
